# Overexpression of chaperonin containing TCP1 subunit 7 has diagnostic and prognostic value for hepatocellular carcinoma

**DOI:** 10.18632/aging.203809

**Published:** 2022-01-24

**Authors:** Xinghua Huang, Huaxiang Wang, Fengfeng Xu, Lizhi Lv, Ruling Wang, Bin Jiang, Tingting Liu, Huanzhang Hu, Yi Jiang

**Affiliations:** 1 The Fuzong Clinical Medical College of Fujian Medical University, Fuzhou, Fujian 350025, PR China; 2 Department of Hepatobiliary Surgery, 900th Hospital of the Joint Logistics Team, Fuzhou, Fujian 350025, PR China; 3 Department of Hepatobiliary and Pancreatic Surgery, Taihe Hospital, Hubei University of Medicine, Shiyan, Hubei 442000, PR China; 4 Graduate School of Fujian University of Traditional Chinese Medicine, Fuzhou, Fujian 350025, PR China

**Keywords:** CCT7, hepatocellular carcinoma, prognosis, diagnosis, spliceosome

## Abstract

Chaperonin containing TCP1 subunit 7 (CCT7) regulates the expression of many tumor-related proteins. We investigated the diagnostic and prognostic value of CCT7 expression for hepatocellular carcinoma (HCC). In datasets from The Cancer Genome Atlas and the Gene Expression Omnibus, CCT7 mRNA levels were greater in HCC tissues than adjacent normal tissues, and these results were validated using immunohistochemistry. In patients with early-stage disease and low alpha-fetoprotein expression, CCT7 expression was still higher in HCC tissues than normal tissues. Receiver operating characteristic curve analyses indicated that CCT7 expression had better diagnostic value than alpha-fetoprotein for HCC patients with early-stage disease and low alpha-fetoprotein expression. The positive predictive value of CCT7 expression was higher than that of alpha-fetoprotein expression. Higher CCT7 mRNA and protein levels were independent risk factors for poorer overall and recurrence-free survival in HCC patients. Greater methylation of the CpG site cg19515186 was associated with better overall survival in HCC patients. Genes co-expressed with CCT7 were upregulated in HCC and associated with poorer overall survival. Gene Ontology, Kyoto Encyclopedia of Genes and Genomes and Gene Set Enrichment Analyses demonstrated that CCT7 expression correlated with spliceosome signaling. These findings demonstrate that CCT7 has diagnostic and prognostic value for HCC.

## INTRODUCTION

Hepatocellular carcinoma (HCC) is one of the most common malignant tumors, and has caused a substantial economic and health burden around the world for many years. According to data released by the American Cancer Society in 2021, HCC is the fifth leading cause of cancer-related mortality, with a five-year survival rate of less than 20% in all stages [1]. The poor prognosis of HCC has mainly been attributed to the low diagnostic rate in the early stage of the disease [2]; indeed, most HCC patients have already missed the opportunity for potentially curative therapeutic interventions by the time they are diagnosed. Currently, liver ultrasound examination and serum alpha-fetoprotein (AFP) analysis are recommended to screen patients with early-stage HCC [3]; however, both of these techniques lack sufficient sensitivity to detect early lesions. Therefore, it is critical to identify more sensitive molecular biomarkers to diagnose early-stage HCC and improve patients’ prognoses.

Chaperonin containing TCP-1 (CCT) is an intracellular chaperonin composed of eight subunits: α, β, γ, δ, ε, ζ, η and θ, which are encoded by *CCT1*, *CCT2*, *CCT3*, *CCT4*, *CCT5*, *CCT6*, *CCT7* and *CCT8*, respectively [4, 5]. CCT promotes the folding of intracellular proteins (mainly cytoskeletal proteins such as tubulin and actin) in the cytoplasm [6]. Since cell division, directed migration and invasion are the main drivers of tumorigenesis and cancer progression, and all these processes depend on the microtubules and actin filaments of the cytoskeleton, CCT activity is fundamentally involved in cancer [5–7]. CCT3 was shown to promote HCC progression by functioning upstream of Yes-associated protein and transcription factor CP2, and thus was suggested as a potential therapeutic target and biomarker for HCC [8]. In addition, Xu and colleagues found that *CCT3* could be a novel therapeutic target associated with breast cancer proliferation and metastasis [9]. High *CCT2*, *CCT5*, *CCT6A* and *CCT7* levels have also been detected in various tumors and associated with patients’ prognoses [10–14]. In bioinformatic analyses, *CCT7* was found to be overexpressed and associated with worse survival in HCC patients; however, the clinical prognostic/diagnostic value and function of this gene have not yet been illustrated [15, 16].

In the present study, we analyzed *CCT7* expression in HCC and adjacent normal tissues from various public databases, and performed immunohistochemical staining of tissues from an HCC cohort. We also evaluated the association of *CCT7* expression with the clinical characteristics and outcomes of HCC patients. We then searched for CpG sites and determined the correlation of *CCT7* methylation with overall survival (OS) in HCC patients. Finally, we performed Gene Ontology (GO), Kyoto Encyclopedia of Genes and Genomes (KEGG) and Gene Set Enrichment Analyses (GSEA) to identify pathways through which CCT7 could contribute to HCC tumorigenesis and progression.

## RESULTS

### CCT7 mRNA expression is significantly upregulated and associated with poorer tumor characteristics in HCC

**Figure 1:**
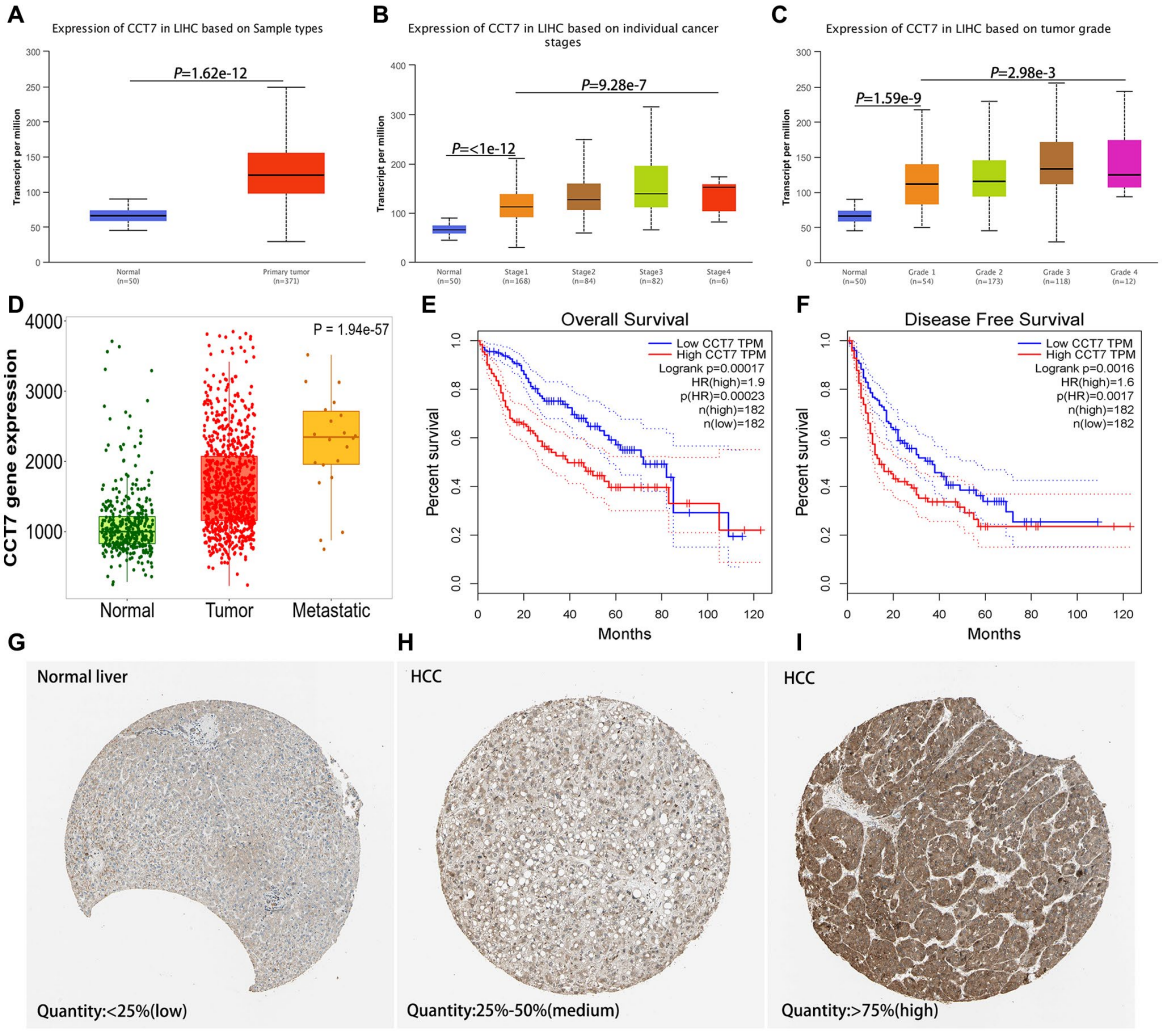
***CCT7* levels in HCC and adjacent normal liver tissues.** (**A**) *CCT7* mRNA levels were significantly greater in HCC than in normal liver tissues. (**B**, **C**) *CCT7* mRNA expression increased incrementally with increasing cancer stages (**B**) and tumor grades (**C**) in HCC tissues. (**D**) *CCT7* mRNA levels were greater in metastatic than in non-metastatic tumor samples. (**E**, **F**) Higher *CCT7* mRNA expression was associated with worse OS (**E**) and RFS (**F**). (**G**–**I**) Representative images from immunohistochemical staining of CCT7 protein expression in normal liver tissues (**G**, expression quantity <25%), low-expression HCC tissues (**H**, expression quantity 25–50%) and high-expression HCC tissues (**I**, expression quantity >75%) from the Human Protein Atlas database.

We analyzed *CCT7* mRNA levels in HCC tissues from The Cancer Genome Atlas (TCGA) database, and visualized the data in the UALCAN database. *CCT7* was significantly upregulated in HCC tissues compared with normal liver samples (Figure 1A). In addition, *CCT7* mRNA levels in HCC samples increased incrementally with increasing cancer stages (Figure 1B) and tumor grades (Figure 1C). In the TNMplot database, *CCT7* mRNA expression was higher in metastatic samples than in non-metastatic tumor samples or normal samples (Figure 1D).

### Higher CCT7 mRNA expression is associated with poorer survival and clinical outcomes in HCC

Next, we analyzed the association between *CCT7* mRNA expression and clinical outcomes using HCC samples from TCGA. Survival curves demonstrated that higher *CCT7* mRNA levels were associated with poorer OS (Figure 1E) and recurrence-free survival (RFS; Figure 1F) in HCC patients. Higher *CCT7* expression was also associated with greater vascular invasion (*P* = 0.015), higher Tumor-Node-Metastasis (TNM) staging (*P* = 0.049), higher tumor grading (*P* = 0.007), greater serum AFP levels (*P* < 0.001), familial cancer history (*P* = 0.011), adjacent hepatic inflammation (*P* = 0.027), fibrosis (*P* = 0.002), recurrence (*P* = 0.037) and poorer survival (*P* = 0.038), but was not associated with age, gender, radiation or pharmaceutical treatment (Table 1).

**Table 1:** Correlation between CCT7 expression and clinical outcomes in HCC in the TCGA database (372 cases).

**Characteristics**			**CCT7 level**		***X* ^2^**	^*^P-Value
		***N***	**High (*n*)**	**Low (*n*)**		
Gender	Male	251	132	119	2.07	0.15
	Female	121	54	67		
Age(years)	>50	301	151	150	0.017	0.895
	<=50	71	35	36		
Vascular invasion	Yes	105	61	44	5.897	0.015
	No	211	92	119		
TNM staging	I/II	269	126	143	3.88	0.049
	III/IV	103	60	43		
Tumor grade	G1/G2	135	80	55	7.267	0.007
	G3/G4	237	106	131		
Serum AFP level(ng/ml)	>400 ng/ml	82	54	28	13.62	<0.001
	<=400 ng/ml	228	96	132		
Family cancer history	Yes	111	68	43	6.409	0.011
	No	207	96	111		
Adjacent hepatic inflammation	Yes	134	75	59	4.859	0.027
	No	121	51	70		
Radiation	Yes	10	7	3	1.644	0.200
	No	362	179	183		
Pharmaceutical	Yes	24	14	10	0.713	0.399
	No	348	172	176		
Fibrosis	Yes	186	106	80	9.558	0.002
	No	123	48	75		
BMI (kg/m^2^)	>=24	111	46	65	4.359	0.037
	<24	226	121	105		
Recurrence	Yes	180	100	80	4.306	0.038
	No	192	86	106		
Survival	Alive	245	105	140	14.646	<0.001

Abbreviations: CCT7: Chaperonin containing TCP1 subunit 7; AFP: alpha fetoprotein; TNM: tumor node metastasis; BMI: body mass index. ^*^P-Value <0.05 were considered statistically significant.

A univariate Cox regression analysis indicated that greater vascular invasion (*P* = 0.003), TNM staging (*P* < 0.001), tumor grading (*P* < 0.001) and *CCT7* mRNA expression (*P* < 0.001) were risk factors for poorer OS in HCC patients. A multivariate Cox regression analysis confirmed that higher TNM staging (hazard ratio [HR] (95% confidence interval [CI]): 2.047 (1.342–3.124); *P* = 0.001), tumor grading (HR (95% CI): 1.808 (1.191–2.744); *P* = 0.005) and *CCT7* mRNA expression (HR (95% CI): 2.031 (1.327–3.110); *P* = 0.001) were independent risk factors for poorer OS in HCC patients (Table 2).

**Table 2:** Univariate and multivariate cox regression analysis of overall survival and recurrence-free survival in TCGA database (372 cases).

**Variables**		**Overall survival**	^*^P-Value	**Recurrence-free survival**	*P-Value
		**HR (95% CI)**		**HR (95% CI)**	
Univariate analysis					
Age(years)	>55 vs. <=55	1.248 (0.786–1.979)	0.348	0.993 (0.689–1.431)	0.969
Gender	Male vs. female	1.224 (0.855–1.754)	0.270	1.019 (0.746–1.392)	0.904
Vascular invasion	Yes vs. no	1.863 (1.242–2.793)	0.003	2.134 (1.523–2.989)	<0.001
TNM staging	I/II vs. III/IV	2.515 (1.771–3.573)	<0.001	1.954 (1.439–2.653)	<0.001
Serum AFP level(ng/ml)	>400 vs <=400	1.441 (0.940–2.210)	0.093	1.337 (0.938–1.905)	0.108
Tumor grade	G1/G2 vs. G3/G4	2.032 (1.431–2.885)	<0.001	1.675 (1.249–2.247)	0.001
Family cancer history	Yes vs. no	1.130 (0.779–1.639)	0.519	0.893 (0.642–1.241)	0.500
Adjacent hepatic inflammation	Yes vs. no	1.415 (0.892–2.244)	0.141	1.213 (0.854–1.725)	0.281
Radiation	Yes vs. no	1.063 (0.392–2.883)	0.904	1.491 (0.699–3.180)	0.302
Pharmaceutical	Yes vs. no	1.100 (0.558–2.168)	0.784	2.098 (1.302–3.379)	0.002
TACE of postoperation	Yes vs. no	0.763 (0.352–1.657)	0.495	1.015 (1.006–1.024)	0.002
Fibrosis	Yes vs. no	0.946 (0.634–1.412)	0.786	1.463 (1.044–2.050)	0.027
BMI (kg/m^2^)	>=24 vs. <24	0.853 (0.570–1.275)	0.438	0.982 (0.710–1.358)	0.912
CCT7	High vs. low	2.143 (1.490–3.081)	<0.001	1.719 (1.279–2.310)	<0.001
Multivariate analysis					
Vascular invasion	Yes vs. no	1.386 (0.912–2.107)	0.126	1.528 (0.998–2.338)	0.049
TNM staging	I/II vs. III/IV	2.047 (1.342–3.124)	0.001	1.790 (1.076–2.714)	0.023
Tumor grade	G1/G2 vs. G3/G4	1.808 (1.191–2.744)	0.005	1.571 (1.045–2.363)	0.030
Pharmaceutical	Yes vs. no			1.486 (0.640–3.449)	0.357
TACE of postoperation	Yes vs. no			2.314 (1.296–4.133)	0.005
Fibrosis	Yes vs. no			1.102 (0.722–1.679)	0.653
CCT7	High vs. low	2.031 (1.327–3.110)	0.001	1.460 (1.039–2.052)	0.029

Abbreviations: CCT7: Chaperonin containing TCP1 subunit 7; AFP: alpha fetoprotein; TNM: tumor node metastasis; BMI: body mass index; HR: hazard ratio; CI: confidential interval. ^*^P-Value <0.05 were considered statistically significant.

For RFS, a univariate Cox regression analysis demonstrated that greater vascular invasion (*P* < 0.001), higher TNM staging (*P* < 0.001), higher tumor grading (*P* = 0.001), pharmaceutical treatment (*P* = 0.002), postoperative transarterial chemoembolization (TACE; *P* = 0.002), fibrosis (*P* = 0.027) and higher *CCT7* mRNA expression (*P* < 0.001) were risk factors for poorer RFS in HCC patients. A multivariate Cox regression analysis confirmed that greater vascular invasion (HR (95% CI): 1.528 (0.998–2.338); *P* = 0.049), higher TNM staging (HR (95% CI): 1.790 (1.076–2.714); *P* = 0.023), higher tumor grading (HR (95% CI): 1.571 (1.045–2.363); *P* = 0.030), postoperative TACE (HR (95% CI): 2.314 (1.296–4.133); *P* = 0.005) and higher *CCT7* mRNA expression (HR (95% CI): 1.460 (1.039–2.052); *P* = 0.029) were independent risk factors for poorer RFS in HCC patients (Table 2).

### Higher CCT7 protein expression correlated with poorer survival and clinical outcomes in a cohort of 118 HCC patients

**Figure 2:**
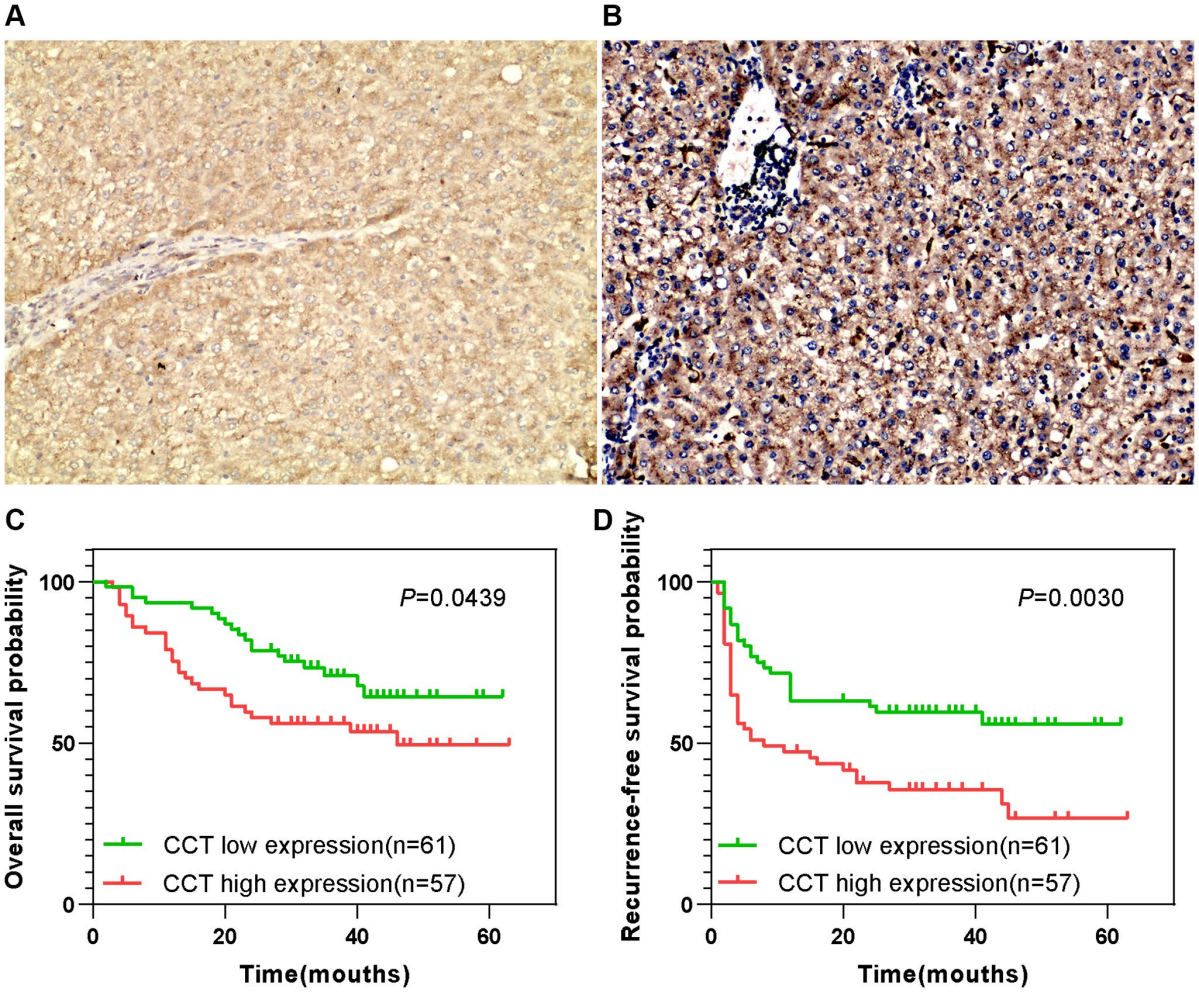
**Prognostic value of CCT7 protein expression in a cohort of 118 HCC patients.** (**A**, **B**) Representative images from immunohistochemical staining of HCC tissues with low (**A**) or high (**B**) CCT7 protein expression (×200 magnification). (**C**, **D**) Higher CCT7 protein expression was associated with worse OS (**C**) and RFS (**D**).

We then evaluated samples from the Human Protein Atlas database, and found that CCT7 protein expression was significantly higher in HCC tissues (Figure 1H and 1I) than in normal liver tissues (Figure 1G). Immunohistochemical staining of tissues from a cohort of 118 HCC patients supported these findings (Figure 2A and 2B). We divided these 118 patients into high and low CCT7 protein expression groups (*n* = 57 and 61, respectively) based on their immunohistochemical scores. Higher CCT7 protein expression was associated with higher TNM staging (*P* = 0.043), serum AFP expression (*P* < 0.001), tumor differentiation (*P* = 0.010), vascular invasion (*P* = 0.029) and recurrence (*P* = 0.005) in HCC patients (Table 3).

**Table 3:** Correlation between CCT7 protein expression and clinical outcomes in HCC patients (*n* = 118).

**Characteristics**			**CCT7 level**		^*^P-Value
		***N***	**High (*n*)**	**Low (*n*)**	
Age (year)	>55	80	40	40	0.593
	<=55	38	17	21	
Gender	Male	103	48	55	0.332
	Female	15	9	6	
Tumor size (cm)	>5cm	67	37	30	0.085
	<=5cm	51	20	31	
TNM staging	I/II	79	33	46	0.043
	III	39	24	15	
Serum AFP level	>400ng/ml	53	36	17	<0.001
	<=400ng/ml	65	21	44	
Tumor location	Left	39	23	16	0.103
	Right	79	34	45	
Tumor differentiation	Low	12	8	4	0.01
	Median	81	43	38	
	High	15	6	19	
Vascular invasion	Yes	52	31	21	0.029
	No	66	26	40	
Tumor encapsulation	Yes	76	32	44	0.070
	No	42	25	17	
Recurrence	Yes	63	38	25	0.005
	No	55	19	36	
Survival	Alive	46	27	19	0.071

Abbreviations: CCT7: Chaperonin containing TCP1 subunit 7; AFP: alpha fetoprotein; TNM: tumor node metastasis. ^*^P-Value <0.05 were considered statistically significant.

A univariate Cox regression analysis revealed that greater tumor sizes (*P* = 0.038), TNM staging (*P* = 0.006), tumor differentiation (*P* = 0.012), vascular invasion (*P* = 0.040) and CCT7 protein expression (*P* = 0.048) were risk factors for poorer OS in HCC patients. A multivariate Cox regression analysis confirmed that greater tumor differentiation (HR (95% CI): 3.232 (1.273–8.208); *P* = 0.014), vascular invasion (HR (95% CI): 2.224 (1.253–3.949); *P* = 0.006) and CCT7 protein expression (HR (95% CI): 1.754 (1.047–2.937); *P* = 0.033) were independent risk factors for poorer OS. For RFS, greater tumor differentiation (HR (95% CI): 2.840 (1.110–7.264); *P* = 0.029), greater vascular invasion (HR (95% CI): 2.106 (1.186–3.426); *P* = 0.010), the absence of tumor encapsulation (HR (95% CI) for tumor encapsulation: 0.303 (0.179–0.511); *P* < 0.001) and higher CCT7 expression (HR (95% CI): 1.695 (1.012–2.839); *P* = 0.045) were both risk factors and independent risk factors for poorer RFS (Table 4). In addition, the durations of OS (Figure 2C) and RFS (Figure 2D) were shorter in the high CCT7 protein expression group than in the low expression group.

**Table 4:** Univariate and multivariate cox regression analysis of overall survival and recurrence-free survival in HCC patients (*n* = 118).

**Variables**		**Overall survival**	^*^P-Value	**Recurrence-free survival**	^*^P-Value
		**HR (95%CI)**		**HR (95%CI)**	
Univariate analysis					
Age (year)	>55	0.799 (0.439–1.456)	0.464	0.755 (0.451–1.264)	0.285
	<=55				
Gender	Male vs. female	0.821 (0.324–2.080)	0.678	1.533 (0.779–3.017)	0.216
Tumor size (cm)	>5 vs. <=5	1.913 (1.038–3.527)	0.038	1.527 (0.921–2.530)	0.101
TNM staging	I/II vs. III	2.262 (1.267–4.039)	0.006	1.573 (0.943–2.624)	0.083
Serum AFP level	>400 vs <=400	1.709 (0.956–3.054)	0.071	1.205 (0.734–1.980)	0.461
Tumor location	Left vs. right	0.825 (0.449–1.514)	0.535	1.182 (0.690–2.024)	0.543
Tumor differentiation	High vs. median/low	4.492 (1.393–14.491)	0.012	4.211 (1.686–10.519)	0.002
Vascular invasion	Yes vs. no	1.877 (1.030–3.419)	0.040	2.359 (1.400–3.974)	0.001
Tumor encapsulation	Yes vs. no	0.837 (0.462–1.515)	0.556	0.258 (0.155–0.431)	<0.001
CCT7	High vs. low	1.810 (1.005–3.259)	0.048	2.062 (1.242–3.422)	0.005
Multivariate analysis					
Tumor size (cm)	>5 vs. <=5	0.832 (0.420–1.651)	0.420		
TNM staging	I/II vs. III	1.462 (0.755–2.829)	0.260		
Tumor differentiation	High vs. median/low	3.232 (1.273–8.208)	0.014	2.840 (1.110–7.264)	0.029
Vascular invasion	Yes vs. no	2.224 (1.253–3.949)	0.006	2.106 (1.186–3.426)	0.010
Tumor encapsulation	Yes vs. no			0.303 (0.179–0.511)	<0.001
CCT7	High vs. low	1.754 (1.047–2.937)	0.033	1.695 (1.012–2.839)	0.045

Abbreviations: CCT7: Chaperonin containing TCP1 subunit 7; AFP: alpha fetoprotein; TNM: tumor node metastasis; HR: hazard ratio; CI: confidential interval. ^*^P-Value <0.05 were considered statistically significant.

### CCT7 is a diagnostic biomarker of HCC

**Figure 3:**
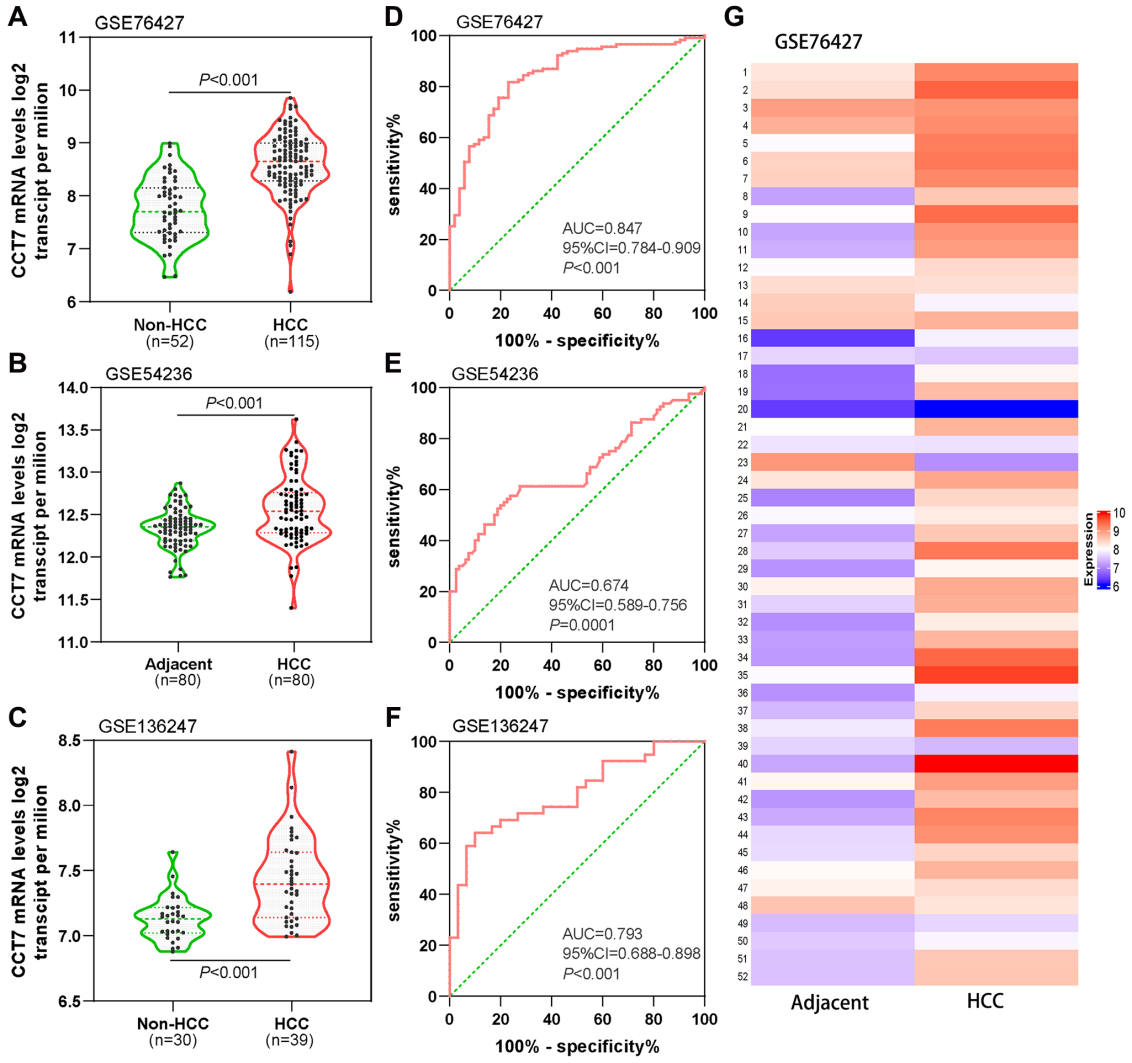
**Diagnostic value of *CCT7* mRNA expression for HCC in the GEO database.*** CCT7* mRNA levels were significantly greater in HCC than in non-HCC tissues in GSE76427 (**A**), GSE54236 (**B**) and GSE136247 (**C**). ROC curves exhibited the good diagnostic significance of *CCT7* mRNA expression for HCC in GSE76427 (**D**), GSE54236 (**E**) and GSE136247 (**F**). (**G**) The heat map shows *CCT7* mRNA expression in 52 paired HCC and corresponding adjacent normal tissues.

Next, we analyzed *CCT7* mRNA expression in the GSE76427 (Figure 3A), GSE54236 (Figure 3B) and GSE136247 (Figure 3C) datasets from the Gene Expression Omnibus (GEO) database. In each dataset, *CCT7* was significantly upregulated in HCC tissues compared with non-HCC tissues (all *P* < 0.001). The corresponding receiver operating characteristic (ROC) curves exhibited good diagnostic significance, with area under the curve (AUC) values of 0.847 (Figure 3D), 0.673 (Figure 3E) and 0.793 (Figure 3F), respectively. Furthermore, a heat map of the GSE76427 dataset revealed that *CCT7* mRNA expression was 90% higher in HCC tissues than in paired adjacent normal liver tissues (Figure 3G).

### CCT7 has a higher positive predictive value (PPV) than AFP for HCC diagnosis

**Figure 4:**
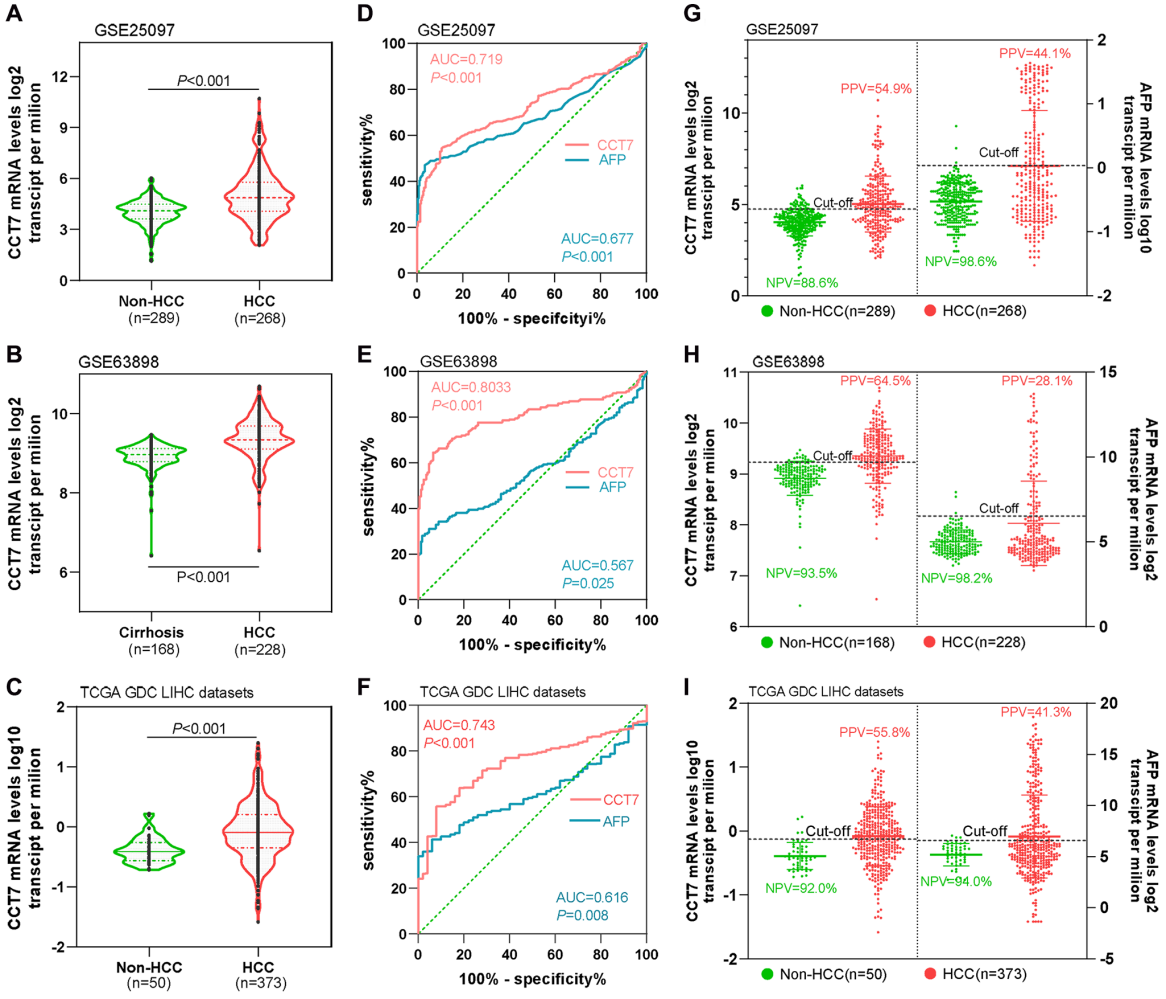
***CCT7* has a higher PPV than *AFP* for HCC diagnosis.*** CCT7* mRNA levels were significantly greater in HCC than in non-HCC tissues in GSE25097 (**A**), GSE63898 (**B**) and TCGA LIHC datasets (**C**). In ROC curve analyses, *CCT7* had a significantly higher AUC than *AFP* in the GSE25097 (0.719 vs. 0.677, **D**), GSE63898 (0.803 vs. 0.567, **E**) and TCGA LIHC datasets (0.743 vs. 0.616, **F**). *CCT7* had a higher PPV than *AFP* in the GSE25097 (54.9% vs. 44.1%, **G**), GSE63898 (64.5% vs. 28.1%, **H**) and TCGA LIHC datasets (55.8% vs. 41.3%, **I**).

We then compared the diagnostic efficiencies of *CCT7* and *AFP* mRNA levels for HCC in the GEO and TCGA databases. *CCT7* mRNA expression was significantly higher in HCC tissues than in normal liver tissues in the GSE25097 (Figure 4A), GSE63898 (Figure 4B) and TCGA liver hepatocellular carcinoma (LIHC) datasets (Figure 4C). ROC curve analyses revealed that *CCT7* had a significantly higher AUC than *AFP* in the GSE25097 (0.719 vs. 0.677, Figure 4D), GSE63898 (0.803 vs. 0.567, Figure 4E) and TCGA LIHC datasets (0.743 vs. 0.616, Figure 4F). The best diagnostic cut-off values for *CCT7* and *AFP* expression were identified based on the sensitivity and specificity values of the ROC curves. We found that *CCT7* had a higher PPV than *AFP* in the GSE25097 (54.9% vs. 44.1%, Figure 4G), GSE63898 (64.5% vs. 28.1%, Figure 4H) and TCGA LIHC datasets (55.8% vs. 41.3%, Figure 4I), even though the two genes had statistically similar negative predictive values (NPVs). These results demonstrated that *CCT7* is more sensitive than *AFP* for the diagnosis of HCC.

### CCT7 has better diagnostic value than AFP in HCC patients with low AFP expression

**Figure 5:**
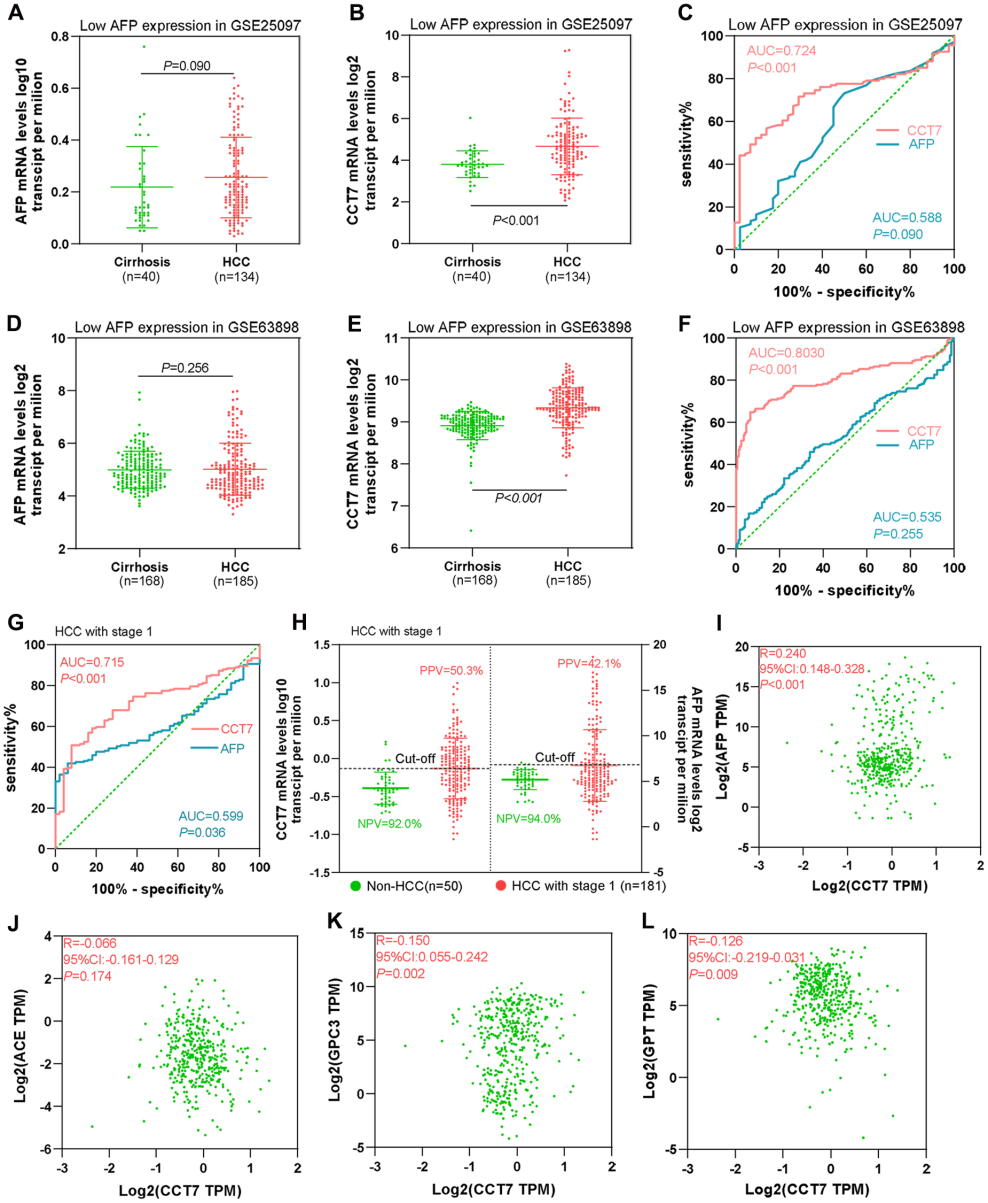
***CCT7* has better diagnostic value than *AFP* for HCC patients with low *AFP* expression and early-stage disease.** (**A**, **B**) *AFP* (**A**) and *CCT7* (**B**) mRNA expression in tissues from cirrhosis patients (*n* = 40) and HCC patients with low *AFP* expression (*n* = 134) in the GSE25097 dataset. (**C**) ROC curve analysis showing the diagnostic value of *AFP* and *CCT7* in HCC patients with low *AFP* expression in the GSE25097 dataset. (**D**, **E**) *AFP* (**D**) and *CCT7* (**E**) mRNA expression in tissues from cirrhosis patients (*n* = 168) and HCC patients with low *AFP* expression (*n* = 185) in the GSE63898 dataset. (**F**) ROC curve analysis showing the diagnostic value of *AFP* and *CCT7* in HCC patients with low *AFP* expression in the GSE63898 dataset. (**G**) ROC curve analysis showing the diagnostic value of *AFP* and *CCT7* in stage 1 HCC patients from TCGA. (**H**) The PPV and NPV of *AFP* and *CCT7* in stage 1 HCC patients from TCGA. (**I**–**L**) The correlations of *CCT7* levels with *AFP* (**I**), *ACE* (**J**), *GPC3* (**K**) and *GPT* (**L**) levels.

AFP is upregulated in no more than 70% of patients with HCC. Thus, we evaluated the diagnostic value of *CCT7* in HCC patients with low *AFP* expression using the GSE25097 and GSE63898 datasets from the GEO database. *AFP* mRNA levels in cirrhosis and HCC patients were similar in the two datasets (Figure 5A, 5D), whereas *CCT7* levels were significantly higher in HCC patients than in cirrhosis patients (Figure 5B, 5E). ROC curve analyses revealed that *AFP* expression had no diagnostic value in either the GSE25097 or the GSE63898 dataset, with AUCs of 0.588 and 0.535, respectively (*P* > 0.05). On the other hand, *CCT7* mRNA expression had significant diagnostic value in both datasets, with AUCs of 0.724 (*P* < 0.001, Figure 5C) and 0.803 (*P* < 0.001, Figure 5F), respectively. These results demonstrated that *CCT7* can be used as an accurate diagnostic biomarker in HCC patients with low *AFP* expression.

### CCT7 is a better diagnostic biomarker than AFP for early-stage HCC patients

Next, we used TCGA to evaluate the diagnostic efficiency of *CCT7* mRNA expression in early-stage HCC patients. ROC curve analyses revealed that *CCT7* had a significantly higher AUC than *AFP* for stage 1 HCC patients (Figure 5G). In addition, *CCT7* mRNA expression had a higher PPV than *AFP* in stage 1 HCC patients from TCGA (50.3% vs. 42.1%), while the two genes had similar NPVs (92.0% vs. 94.0%, Figure 5H). We also investigated the correlation of *CCT7* levels with those of previously identified diagnostic biomarkers [17–19], and obtained the following correlation coefficients: *AFP*, r = 0.240; angiotensin converting enzyme (*ACE*), r = 0.066; glypican 3 (*GPC3*), r = −0.150; and glutamic-pyruvic transaminase (*GPT*), r = −0.126 (Figure 5I–5L). These results suggested that *CCT7* expression could be used as an independent diagnostic biomarker for HCC patients.

### Dysregulation of CCT7 expression is associated with DNA methylation status in HCC patients

**Figure 6:**
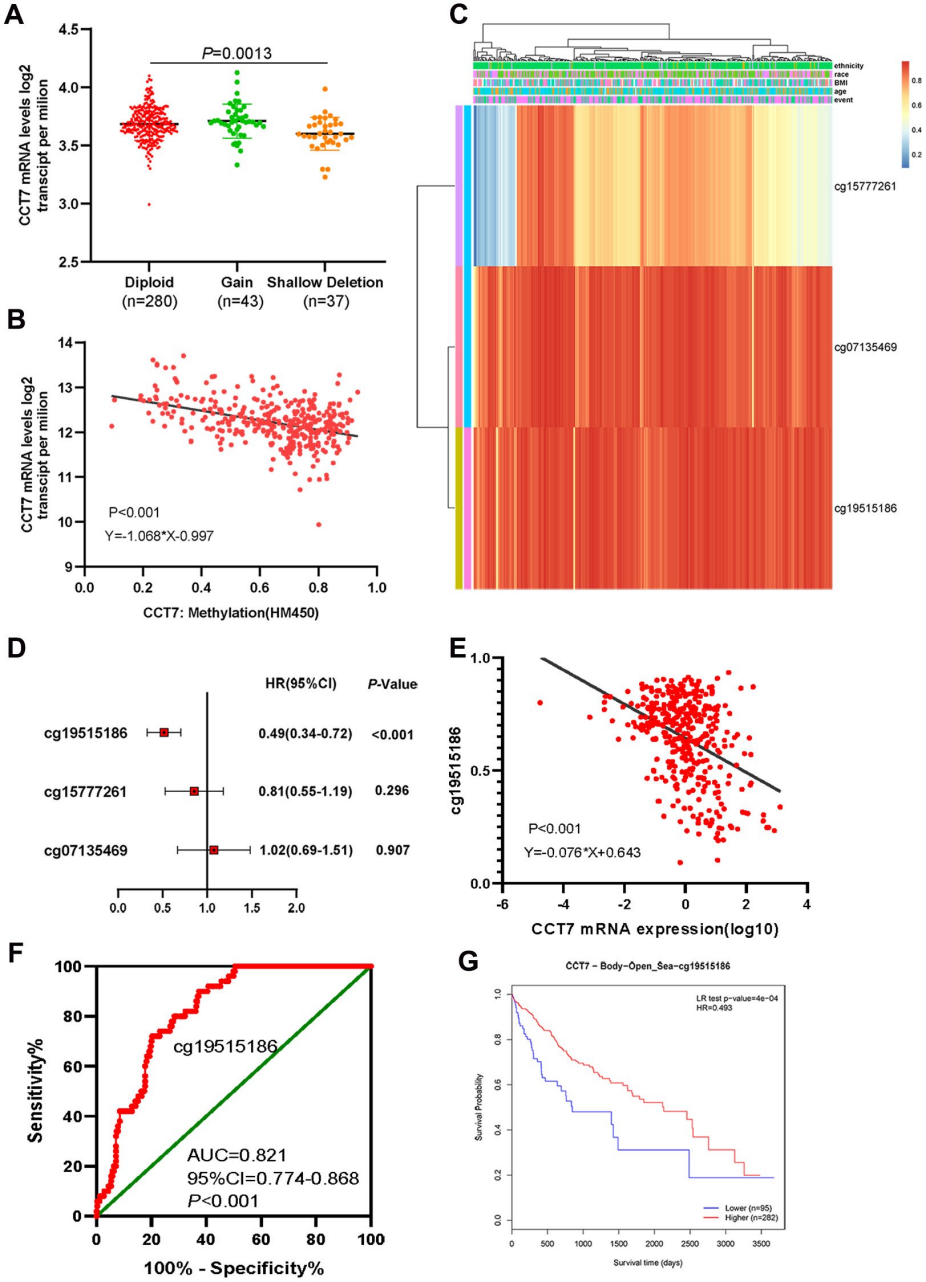
**Dysregulated *CCT7* expression is associated with DNA methylation status in HCC patients.** (**A**) *CCT7* mRNA expression in different copy number groups. (**B**) The correlation between *CCT7* mRNA expression and DNA methylation status. (**C**) The heat map shows *CCT7*-related methylated CpG sites in HCC. (**D**) The forest map shows the correlation between CpG site methylation and survival times in HCC patients. (**E**) The correlation between *CCT7* mRNA expression and cg19515186 methylation status. (**F**) ROC curve analysis showing the significant diagnostic value of cg19515186 methylation status for HCC in TCGA. (**G**) Survival analysis showing that higher methylation of cg19515186 was associated with better OS in HCC patients.

Our data indicated that *CCT7* mRNA expression was frequently upregulated in HCC patients, and further analyses revealed that this dysregulation was associated with copy number alterations (Figure 6A). When we evaluated Illumina Human Methylation 450 datasets in TCGA, we found that *CCT7* mRNA expression was negatively associated with DNA methylation status (Figure 6B). Using the MethSurv database, we identified three *CCT7*-related methylated CpG sites in HCC: cg15777261, cg07135469 and cg19515186 (Figure 6C). One of these CpG sites (cg19515186) was associated with the survival times of HCC patients (*P* < 0.001, HR (95% CI): 0.49 (0.34–0.72); Figure 6D). A correlation analysis also revealed that the methylation status of cg19515186 was negatively associated with *CCT7* mRNA expression (Figure 6E). Furthermore, ROC curve analysis indicated that the methylation status of cg19515186 had significant diagnostic value for HCC in TCGA (AUC = 0.821, *P* < 0.001, Figure 6F). We also performed a survival analysis, which revealed that higher methylation of cg19515186 was associated with better OS in HCC patients (Figure 6G). These results demonstrated that *CCT7* expression is associated with the DNA methylation status of HCC patients.

### Genetic alterations of CCT7 are associated with poorer survival in HCC patients

**Figure 7:**
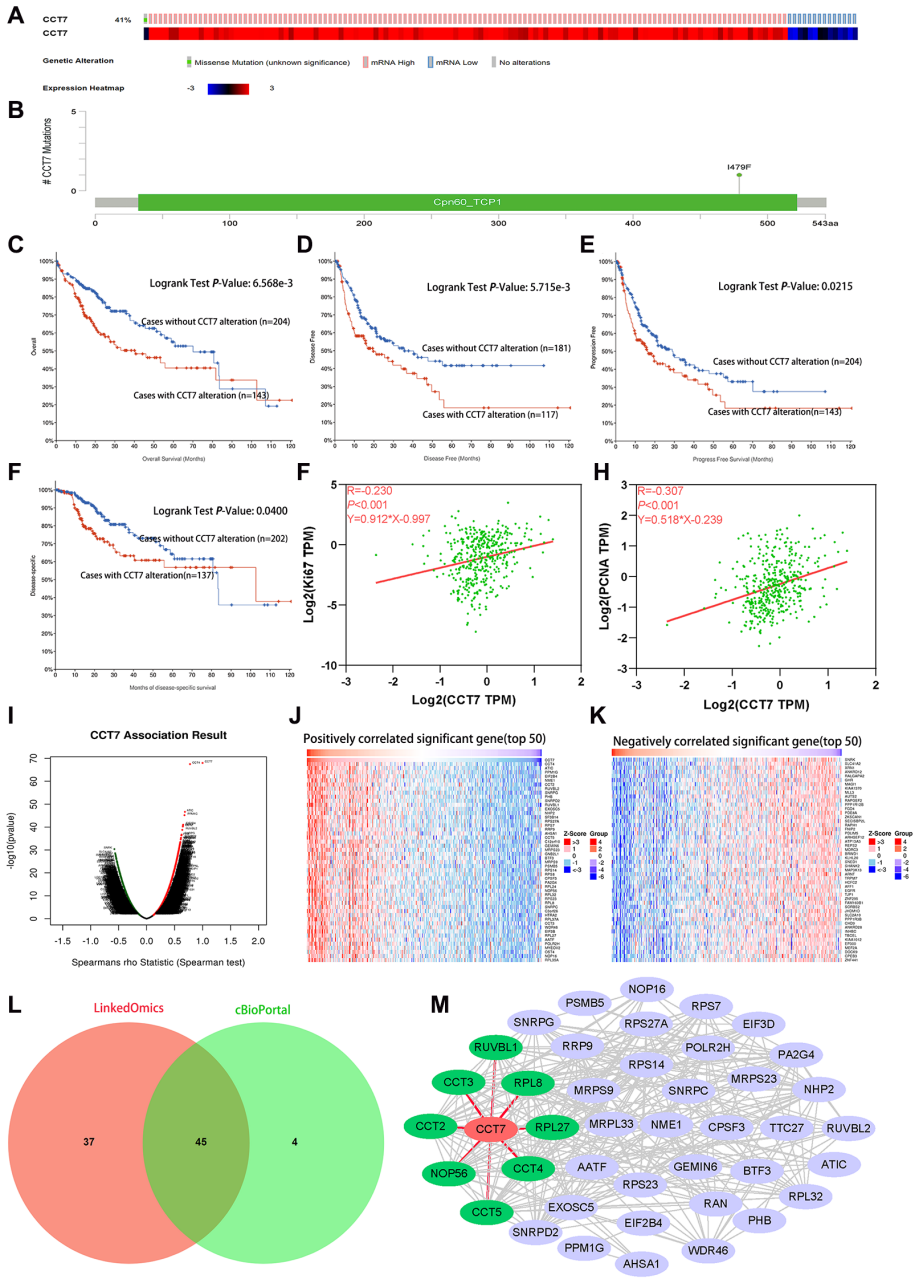
**Genetic alterations of *CCT7* are associated with poorer survival in HCC patients.** (**A**) *CCT7* was genetically altered in 143 patients (41%) from a cohort of 348 HCC patients. (**B**) A mutational hotspot of 1479F/Missense was found in 104 patients. (**C**–**F**) Survival analyses showing that HCC patients with *CCT7* alterations had poorer OS (**C**), disease-free survival (**D**), progression-free survival (**E**) and disease-specific survival (**F**) than those without. (**G**, **H**) Spearman’s correlation analysis revealing that *CCT7* mRNA expression correlated positively with Ki67 (**G**) and PCNA (**H**) expression. (**I**–**K**) *CCT7* expression-associated target gene analysis in the LinkedOmics database. (**I**) Volcano chart exhibiting genes with significant positive/negative correlations with *CCT7* expression. (**J**) Top 50 genes that were positively associated with *CCT7* expression. (**K**) Top 50 genes that were negatively associated with *CCT7* expression. (**L**) Venn plot showing the overlapping genes from the LinkedOmics and cBioPortal databases with Spearman’s values greater than 0.55. (**M**) PPI network for 45 genes co-expressed with CCT7. CCT2, CCT3, CCT4, CCT5, NOP56, RPL8, RPL27 and RUVBL1 were found to interact with CCT7.

We then used the cBioPortal database to search for genetic alterations of *CCT7* in a cohort of 348 HCC patients. We detected genetic alterations of *CCT7* in 143 (41%) of the queried patients, including 1 case of a missense mutation, 14 cases of low expression and 128 cases of high expression (Figure 7A). In addition, we found a mutational hotspot of 1479F/Missense in 104 samples (Figure 7B). Somatic mutations of *CCT7* were observed in 0.3% of the patients. Kaplan-Meier survival analyses demonstrated that, compared with HCC patients without *CCT7* alterations, HCC patients with *CCT7* alterations had poorer OS (*P* = 6.568e-03, Figure 7C), disease-free survival (*P* = 5.715e-03, Figure 7D), progression-free survival (*P* = 2.150e-02, Figure 7E) and disease-specific survival (*P* = 4.0e-02, Figure 7F). A Spearman’s correlation analysis revealed that *CCT7* mRNA expression correlated positively with other prognostic biomarkers in HCC patients (Ki67: r = 0.230, *P* < 0.001; proliferating cell nuclear antigen [PCNA]: r = 0.307, *P* < 0.001; Figure 7G and 7H).

### Analysis of genes co-expressed with CCT7 in HCC patients

Subsequently, we used LIHC datasets from the LinkedOmics and cBioPortal databases to identify genes that correlated with *CCT7* in their expression. A volcano plot and heat map were used to depict the 11319 genes that correlated negatively with *CCT7* and the 8670 genes that correlated positively with *CCT7* in the LinkedOmics database (Figure 7I–7K). We selected overlapping genes from the two databases with Spearman’s values greater than 0.55, and thus identified 45 genes that were co-expressed with *CCT7* (Figure 7L).

**Figure 8:**
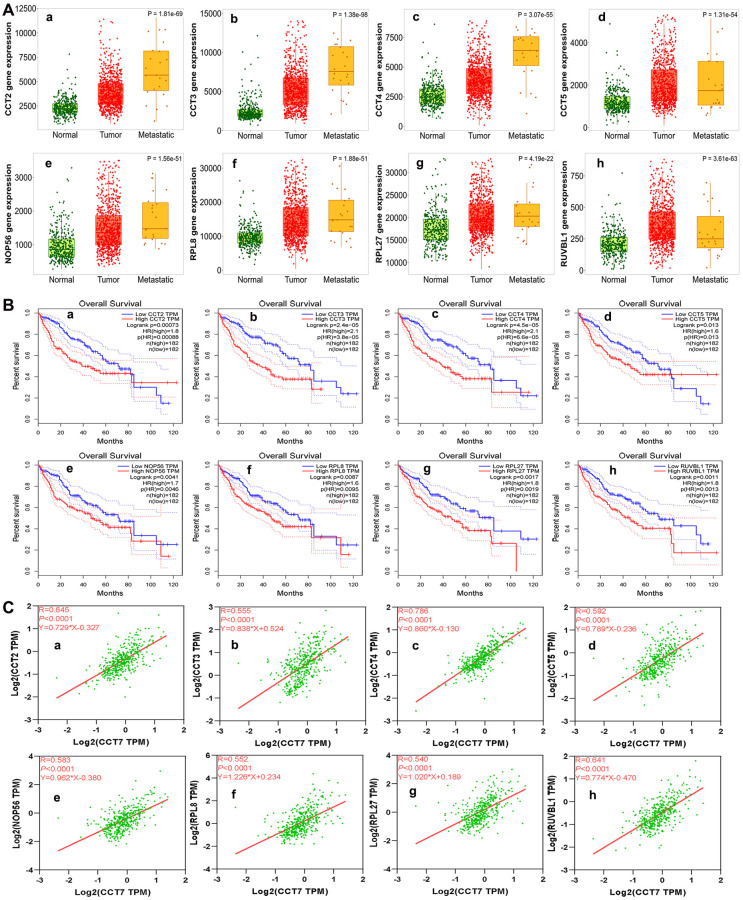
**Analysis of genes co-expressed with *CCT7* in HCC patients.** (**A**) The mRNA levels of *CCT2* (**a**), *CCT3* (**b**), *CCT4* (**c**), *CCT5* (**d**), *NOP56* (**e**), *RPL8* (**f**), *RPL27* (**g**) and *RUVBL1* (**h**) in normal, tumor and metastatic tissues. (**B**) Survival analysis showing associations of *CCT2* (**a**), *CCT3* (**b**), *CCT4* (**c**), *CCT5* (**d**), *NOP56* (**e**), *RPL8* (**f**), *RPL27* (**g**) and *RUVBL1* (**h**) mRNA levels with the OS of HCC patients (all *P* < 0.05). (**C**) Correlation of *CCT7* levels with *CCT2* (**a**), *CCT3* (**b**), *CCT4* (**c**), *CCT5* (**d**), *NOP56* (**e**), *RPL8* (**f**), *RPL27* (**g**) and *RUVBL1* (**h**) levels.

Next, we used the Search Tool for the Retrieval of Interacting Genes (STRING) database to identify significant interactions among CCT7 and its 45 co-expressed genes, based on confidence scores greater than 0.9. A protein-protein interaction (PPI) network with 42 nodes and 288 edges was constructed and visualized using Cytoscape software. Eight proteins (CCT2, CCT3, CCT4, CCT5, nucleolar protein 56 [NOP56], ribosomal protein L8 [RPL8], RPL27 and RuvB-like AAA ATPase 1 [RUVBL1]) directly interacted with CCT7 in the PPI network (Figure 7M). Using the TNMplot database, we found that the mRNA levels of these eight genes increased incrementally from normal tissues to HCC tissues to metastatic HCC tissues (Figure 8A a–h). When we performed a survival analysis in the Gene Expression Profiling Interactive Analysis (GEPIA) database, we found that higher levels of these eight genes were associated with poorer OS in HCC patients (Figure 8B a–h). The significant positive correlations between the levels of *CCT7* and these eight genes were validated in TCGA (Figure 8C a–h).

### High CCT7 expression correlates with the spliceosome signaling pathway

**Figure 9:**
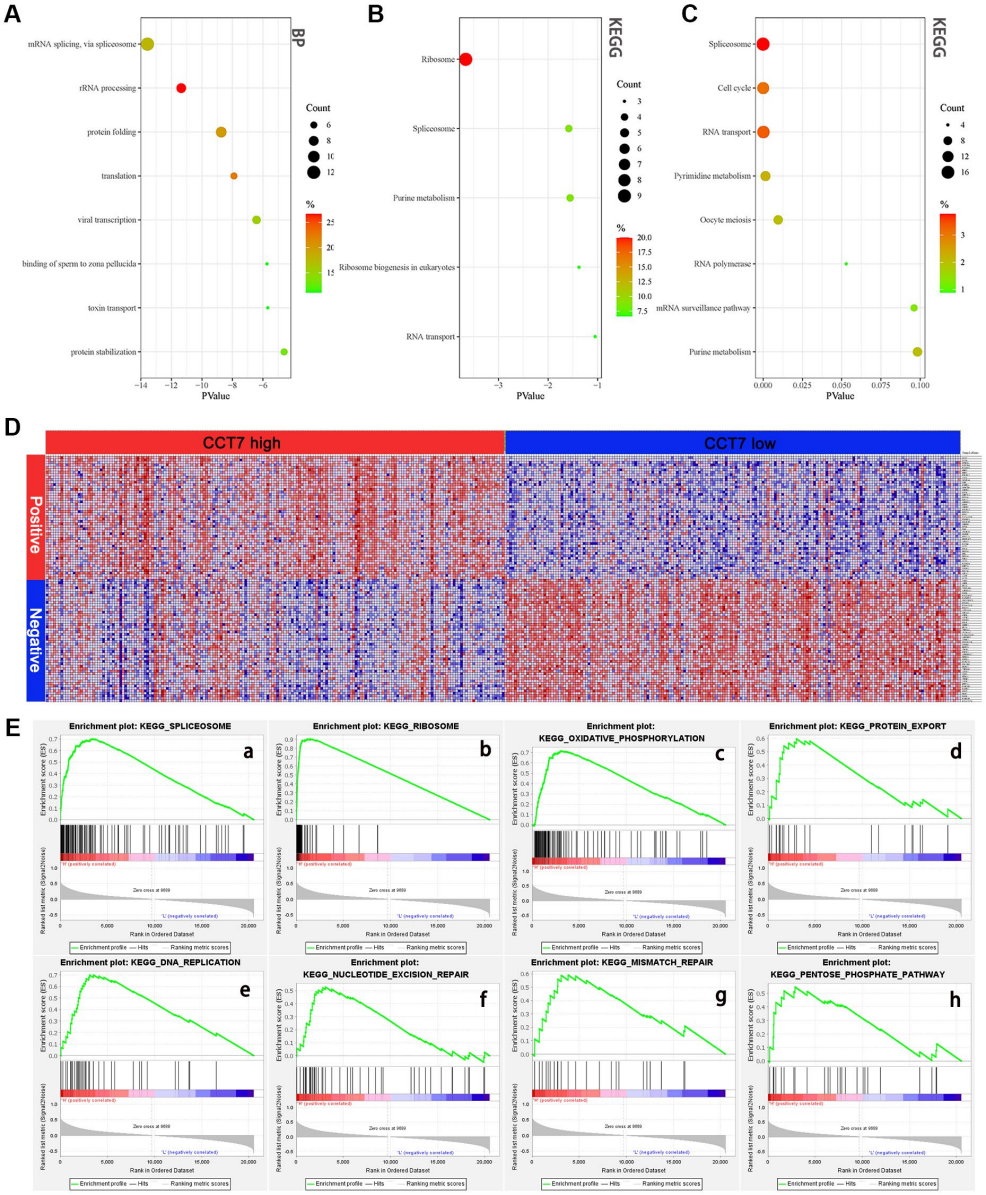
**High *CCT7* expression is associated with spliceosome gene expression in HCC patients.** (**A**, **B**) The 45 genes co-expressed with *CCT7* in HCC tissues, based on the GO Biological Process analysis (**A**) and KEGG pathway analysis (**B**). (**C**) The most significant survival-associated genes in HCC tissues, based on KEGG pathway analysis of data from the GEPIA database. (**D**) Heat map showing the median mRNA levels of genes co-expressed with *CCT7* in HCC tissues in the GSEA. (**E**) The main enriched KEGG pathways of *CCT7* based on GSEA. SPLICEOSOME (**a**), RIBOSOME (**b**), OXIDATIVE PHOSPHORYLATION (**c**), PROTEIN EXPORT (**d**), DNA REPLICATION (**e**), NUCLEOTIDE EXCISION PEPAIR (**f**), MISMATCH REPAIR (**g**), PENTOSE PHOSPHATE PATHWAY (**h**).

We then used the Database for Annotation, Visualization and Integrated Discovery (DAVID) to perform GO and KEGG analyses on the 45 genes co-expressed with *CCT7*. In the GO Biological Process analysis, the co-expressed genes were most enriched in ‘mRNA splicing via spliceosome’, ‘rRNA processing’, ‘protein folding’ and ‘translation’ (Figure 9A). We also performed GO enrichment analyses for Cellular Component and Molecular Function (Supplementary Figure 1). In the KEGG analysis, the ‘ribosome’, ‘spliceosome’, ‘purine metabolism’ and ‘ribosome biogenesis in eukaryotes’ were greatly enriched (Figure 9B). We then analyzed the KEGG pathways of the enriched genes that were most relevant to the survival of HCC patients in the GEPIA database (listed in Supplementary Table 1). The KEGG analysis revealed that the spliceosome pathway correlated significantly with the prognosis of HCC patients (Figure 9C).

**Figure 10:**
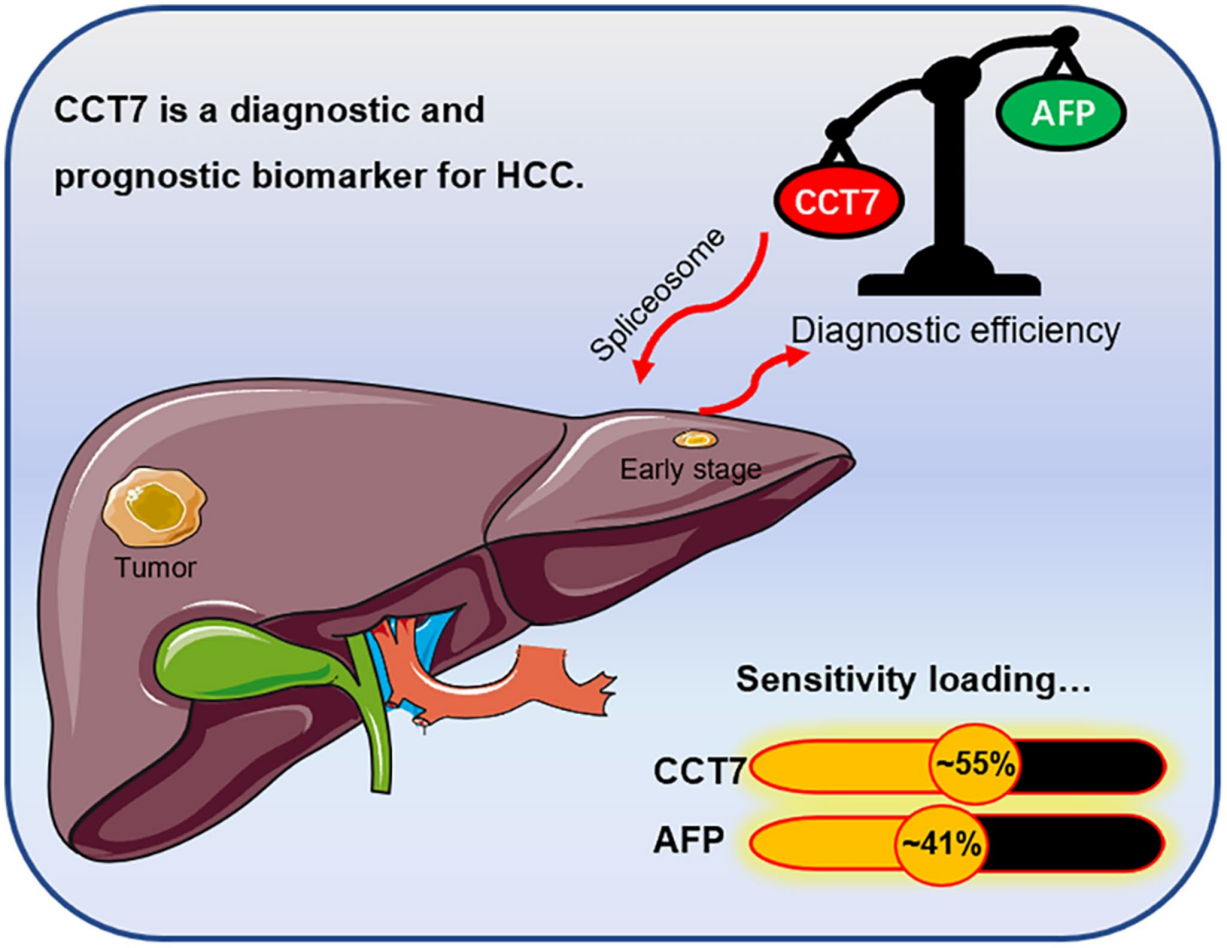
**Graphical representation of the diagnostic sensitivity of *CCT7* and *AFP* in HCC patients from TCGA.*** CCT7* exhibited significantly greater diagnostic value than *AFP*. *CCT7* functions as an oncogene that promotes HCC tumorigenesis and progression through the spliceosome signaling pathway.

To further investigate the potential pathways whereby CCT7 promotes the tumorigenesis and development of HCC, we performed a GSEA. The genes co-expressed with *CCT7* in HCC tissues are shown in Figure 9D, and the top eight differentially regulated pathways are shown in (Figure 9E a–h). The normalized enrichment score (NES) for the spliceosome signaling pathway was 2.09, demonstrating that this pathway correlated positively with HCC tumorigenesis and progression. These results revealed that *CCT7* mRNA expression correlated positively with the spliceosome signaling pathway in HCC (Figure 10).

## DISCUSSION

Numerous studies have demonstrated that various CCT subunits significantly induce tumor proliferation and migration in various cancers [10, 20, 21]. Huang et al. reported that CCT8 was upregulated in HCC and promoted cancer cell proliferation [22]. In addition, Zhang et al. demonstrated that CCT3 promoted HCC cell proliferation by facilitating mitosis and suppressing apoptosis [23]. CCT4, CCT6A and CCT6B were determined to have diagnostic and prognostic value for HCC [16]. Although *CCT7* was found to be highly expressed in HCC tissues in a bioinformatic analysis [15], the present study is the first systematic investigation of the diagnostic value, clinical significance and function of *CCT7* in HCC.

We found that *CCT7* mRNA expression was significantly greater in HCC samples than in normal tissues. *CCT7* expression increased incrementally with increasing cancer stages and tumor grades, and was greater in metastatic than in non-metastatic tumor samples. Our correlation analyses suggested that higher *CCT7* expression was associated with worse clinicopathological features and was an independent risk factor for worse OS and RFS. CCT7 protein expression was also significantly upregulated in HCC tissues compared with adjacent normal tissues, and higher CCT7 protein levels were associated with poorer clinicopathological features. These results demonstrated that *CCT7* can be used as a diagnostic and prognostic biomarker of HCC.

The current gold-standard biomarker for diagnosing HCC is AFP [24, 25]; however, its sensitivity is not satisfactory, especially in AFP-negative patients. We thus compared the diagnostic efficiencies of *CCT7* and *AFP* using independent datasets from the GEO and TCGA databases. In ROC curve analyses, *CCT7* had a higher AUC than *AFP*. *CCT7* also exhibited a better PPV than *AFP*, and was highly expressed in HCC patients with low *AFP* expression, suggesting that *CCT7* has greater diagnostic significance than *AFP* in such patients. Moreover, in patients with early-stage HCC, *CCT7* had a better AUC (0.715 vs. 0.599) and PPV (50.3% vs. 42.1%) than *AFP* in TCGA. It should be noted that our results were based on an HCC tissue mRNA assay rather than a serum analysis. Nevertheless, a previous study demonstrated that serum CCT3 was a potential biomarker of liver cancer with a better diagnostic capacity than AFP in certain regards [8]. Further experimental research is needed to evaluate the clinical diagnostic applicability of *CCT7* for HCC patients.

DNA methylation is dysregulated in virtually all forms of cancer, and has been shown to silence a broad range of genes in different cancer types [26–28]. We found that the upregulation of *CCT7* was associated with the hypomethylation of the CpG site cg19515186. In addition, greater methylation of cg19515186 was associated with better OS in HCC patients. These results demonstrated that *CCT7* expression is associated with DNA methylation status in HCC patients.

Next, we investigated the function of *CCT7* in HCC by performing GO, KEGG and GSEA studies. Each of these analyses revealed that *CCT7* was involved in the spliceosome signaling pathway. Spliceosome signaling contributes significantly to the tumorigenesis and progression of several types of tumors [29–32], and previous research has suggested that CCT7 promotes the progression of endometrial cancer through this pathway [33]. Interestingly, in our study, the genes enriched in the spliceosome pathway were among those most relevant to the survival of HCC patients in the GEPIA database. Thus, *CCT7* seems to function as an oncogene that enhances HCC tumorigenesis and progression through the spliceosome signaling pathway.

In previous studies, siRNA-induced depletion of *CCT3* and *CCT8* was found to block S-phase entry and inhibit the proliferation of HCC cells [22, 34]. In addition, the knockdown of *CCT3* was shown to inhibit the activation of signal transducer and activator of transcription 3. Xu and colleagues reported that *CCT5* was the direct target of miR-139-5p and promoted the progression of HCC through the spliceosome pathway [35]. To the best of our knowledge, no previous studies have described the molecular mechanisms of CCT7 activity in HCC; however, consistent with the known functions of CCT3 and CCT5 in HCC, our study revealed that CCT7 promotes the progression of HCC through the spliceosome pathway. Therefore, it is reasonable to hypothesize that CCT7 regulates signal transducer and activator of transcription 3, thus influencing the cell cycle. This possibility requires further research.

There has been increasing interest in the therapeutic potential of splicing modulation in spliceosome-mutant cancers [36, 37]. Seiler et al. found that the orally available small-molecule splicing modulator H3B-8800 could potently kill spliceosome-mutant epithelial and hematologic tumor cells [38]. Our results indicated that 41% of queried HCC patients exhibited genetic alterations in *CCT7*, and such alterations predicted a poor prognosis. Thus, drugs designed to address *CCT7* alterations could be useful for the treatment of HCC.

In summary, the present study demonstrated that CCT7 mRNA and protein levels were significantly greater in HCC tissues than in adjacent normal liver tissues. Higher *CCT7* levels were associated with poorer clinical outcomes and prognoses. *CCT7* was found to be an effective diagnostic and prognostic biomarker for HCC patients, especially low-*AFP*-expressing and early-stage patients. The upregulation of *CCT7* was associated with the hypomethylation of the CpG site cg19515186. *CCT7* was found to function as an oncogene that promotes HCC tumorigenesis and progression through the spliceosome signaling pathway.

## MATERIALS AND METHODS

### Gene expression analysis

We analyzed *CCT7* mRNA levels using the UALCAN database [39], which visualizes data from TCGA [40]. The TNMplot database (https://tnmplot.com/analysis/) was used to compare *CCT7* mRNA levels between metastatic and non-metastatic tumors [41]. The GEPIA database (http://gepia.cancer-pku.cn/) was used to analyze the association of *CCT7* mRNA expression with OS and RFS [42]. The Human Protein Atlas database (https://www.proteinatlas.org/) was used to evaluate CCT7 protein expression [43]. We also used GEO (GSE76247, GSE54236, GSE136247, GSE25097 and GSE63898) [44] and TCGA datasets to analyze the mRNA levels and compare the diagnostic efficiencies of *CCT7* and *AFP*.

### Prognostic value analysis using CCT7 mRNA expression and clinicopathological data from TCGA

We downloaded RNAseq data and clinical characteristics from TCGA to determine the relationship between *CCT7* expression and clinical outcomes in HCC patients. To evaluate the prognostic value of *CCT7*, we divided 372 HCC patients into high and low expression groups based on their *CCT7* mRNA levels. We defined OS as the time interval between surgery and death or between surgery and the last observation point. We defined RFS as the time interval between the date of surgery and the date of diagnosis of any type of recurrence [45].

### Prognostic value analysis using CCT7 protein expression and clinicopathological data from a cohort of 118 HCC patients

To further investigate the association of CCT7 protein expression with clinical outcomes, we performed immunohistochemical staining using 118 HCC tissues and paired adjacent normal liver tissues. The tissues were collected from patients who underwent hepatectomies at the 900^th^ Hospital of the Joint Logistics Team from February 2013 to November 2014. The shortest follow-up time was five years. Follow-up data were obtained through re-examinations, telephone calls and the Social Security Death Index. Liver function and tumor stages were assessed using the Child-Pugh classification and the 2010 International Union Against Cancer TNM classification system, respectively [46, 47]. Patients met the inclusion criteria if they had one or more lesions confined to one liver lobe, had no distant metastases, had not received chemotherapy, TACE or immunotherapy before surgery, and were confirmed to have HCC based on postoperative pathology. This study was performed in accordance with the principles of the Declaration of Helsinki, and was approved by the Human Research Ethics Committee of the 900^th^ Hospital of the Joint Logistics Team (Fuzhou, China). All participants provided written informed consent before surgery and specimen collection.

### Immunohistochemical analysis

The 118 HCC specimens were cut into 4-μm sections and fixed on glass slides for microscopy. The tissue sections on the slides were then deparaffinized and rehydrated using gradient concentrations of malondialdehyde and ethanol. Next, the slides were immersed in boiling Tris/ethylenediaminetetraacetic acid (pH 9.0) for 20 minutes for antigen retrieval. The slides were subsequently immersed in 3% H_2_O_2_ for 10 minutes to inhibit endogenous peroxidase. Then, the slides were incubated with a primary antibody against CCT7 (1:250; 15994-1-AP; Proteintech, Wuhan, China), followed by the secondary antibody (1:50,000; KIT-5010, anti-rabbit/mouse IgG; Maixin Biotechnology Development Co., Ltd., Fuzhou, China), and were washed three times with phosphate-buffered saline. Finally, the sections were stained with 3,3'-diaminobenzidine and substrate chromogen (Dako) for 2 minutes at room temperature and then counterstained with hematoxylin for 40 seconds. Slides incubated only with the secondary antibody without the primary antibody were used as the negative control. The immunohistochemical staining was assessed by two separate pathologists who were blinded to patients’ information. CCT7 protein expression was assessed on the following five-point scale: 0, no positive cells; 1, <25% positive cells; 2, 26–50% positive cells; 3, 51–75% positive cells; and 4, >75% positive cells.

### DNA methylation and genetic alteration of CCT7 in HCC

We used RNAseq and Illumina Human Methylation 450 datasets from TCGA (https://xenabrowser.net/datapages/) [48] to obtain gene expression and DNA methylation data, respectively. Then, we analyzed the correlation between *CCT7* mRNA expression and DNA methylation. To identify CpG sites that influenced mRNA expression, we used MethSurv, a web tool that performs multivariable survival analyses using DNA methylation data [49]. We investigated genetic alterations and mutational hotspots in *CCT7* using the LIHC (TCGA, Firehose Legacy) dataset from the cBioPortal database (http://www.cbioportal.org/) [50]. Then, we analyzed the correlation between genetic alterations in *CCT7* and the prognoses of HCC patients.

### GO and KEGG enrichment analyses and PPI network construction

We used LIHC datasets from the cBioPortal and LinkedOmics databases [51] to identify genes that correlated with *CCT7* in their expression. Overlapping genes from cBioPortal and LinkedOmics with Spearman’s correlation values greater than 0.55 were selected as the co-expressed genes of *CCT7*. Next, the Functional Annotation Tool in DAVID was used to perform GO and KEGG enrichment analyses on these genes, in order to explore their potential involvement in HCC tumorigenesis and progression [52]. We also constructed a PPI network from these genes in the STRING database, and visualized the network using Cytoscape software (Version 3.7.2) [53]. For the GO and KEGG enrichment analyses, *P* < 0.05 and a false discovery rate <0.25 were considered statistically significant.

### GSEA

Normalized gene expression data from 373 HCC samples were downloaded from TCGA, and the samples were divided into high and low expression groups based on the median *CCT7* expression value. Then, GSEA software (Version 4.1.2) was used to perform KEGG enrichment analyses. In this process, "c2.cp.kegg.v7.0.symbols.gmt" was selected as the functional gene set, and the number of permutations was set to 1000. The default settings were used for all other parameters. Pathways and genes with normal *P*-values <0.05 and false discovery rate *q*-values <0.25 were considered significantly enriched.

### Statistical analysis

Statistical analyses were performed and figures were generated using GraphPad Prism 6.0 (GraphPad Software, Inc., San Diego, CA, USA). The associations between *CCT7* mRNA levels and clinicopathological characteristics were analyzed using two-tailed Student’s t-tests, Fisher’s exact tests or Wilcoxon’s tests. Pearson’s chi-square test was used to compare categorical variables. The Kaplan-Meier method was used with the log-rank test to generate OS and RFS curves. Univariate and multivariate analyses with Cox’s proportional regression models were used to predict the risk factors and independent risk factors affecting survival in HCC patients. The AUCs of ROC curves were used to estimate the diagnostic value of *CCT7* mRNA expression and cg19515186 DNA methylation for HCC. *P* < 0.05 was defined as a statistically significant difference unless otherwise stated.

### Availability of data and materials

All data generated or analyzed during this study are included in this published article.

### Ethics approval and consent to participate

This study was performed according to the relevant medical ethics regulations and approved by the Human Research Ethics Committee of the 900th Hospital of the Joint Logistics Team (Fuzhou, China). All participants gave written informed consent prior to surgery and specimen collection.

## Supplementary Materials

Supplementary Figure 1

Supplementary Table 1
