# Supplementary material for: Overexpression of chaperonin containing TCP1 subunit 7 has diagnostic and prognostic value for hepatocellular carcinoma
Source: Aging (Albany NY). 2022 Jan 24;14(2):747–69. doi: 10.18632/aging.203809 (PMC8833116; doi:10.18632/aging.203809)
Supplement: Supplementary Figure 1 [file aging-14-203809-s001.pdf]

SUPPLEMENTARY FIGURE

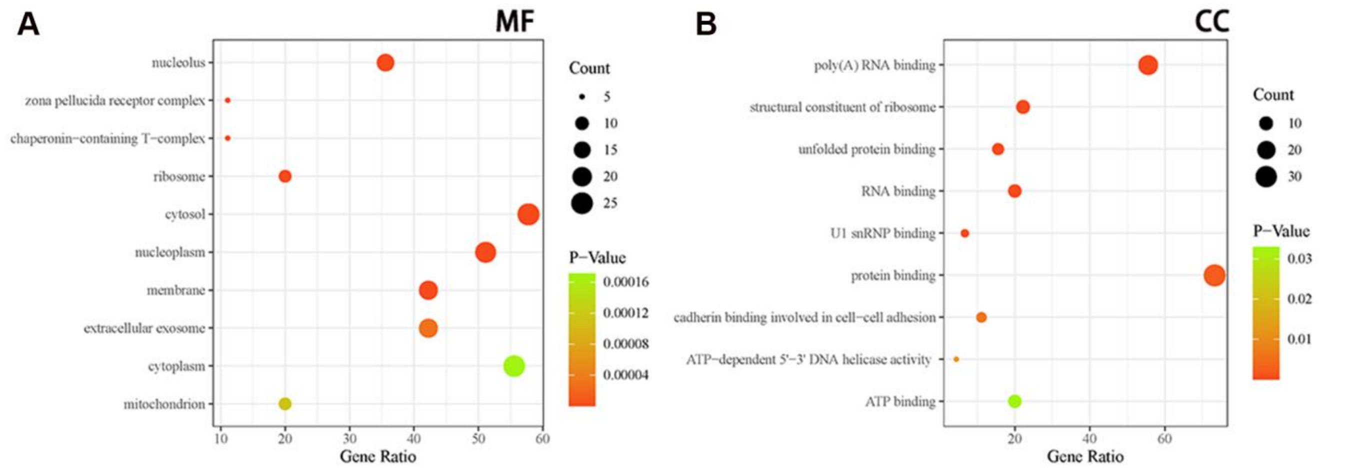

Supplementary Figure 1. (**A**) The 45 co-expressed genes of CCT7 in the HCC tissues based on the molecular functions (MF)-GO analysis are shown. (**B**) The 45 co-expressed genes of CCT7 in the HCC tissues based on the cellular component (CC)-GO analysis are shown.
